# (η^6^-Benzene){2-[2-(*tert*-butyl­sulfan­yl)phenyl]pyridine-κ^2^ *N*,*S*}chlorido­ruthenium(II) hexa­fluorido­phosphate

**DOI:** 10.1107/S1600536810049810

**Published:** 2010-12-11

**Authors:** Masakazu Hirotsu, Akira Yogi, Isamu Kinoshita

**Affiliations:** a Graduate School of Science, Osaka City University, Sumiyoshi-ku, Osaka 558-8585, Japan

## Abstract

In the title compound, [RuCl(C_6_H_6_)(C_15_H_17_NS)]PF_6_, the cation adopts a three-legged piano-stool structure around the Ru(II) atom with an η^6^-benzene ligand, a chloride ligand and a 2-[2-(*tert*-butyl­sulfan­yl)phen­yl]pyridine (btppy) ligand. The btppy ligand acts as a *N*,*S*-bidentate ligand, forming a six-membered ring, which has an envelope conformation. The S—Ru—N bite angle is 86.76 (9)°, and the dihedral angle between the pyridine and benzene rings in btppy is 39.8 (2)°. The unit cell contains two pairs of racemic diastereomers with (*S*_Ru_,*S*_S_) and (*R*_Ru_,*R*_S_) configurations, in which the *tert*-butyl group on the coordin­ated S atom is distant from the η^6^-benzene ligand.

## Related literature

For general background to the use of chiral *N,S*-bidentate ligands in asymmetric allylic substitution reactions, see: Mellah *et al.* (2007 ▶). For the synthesis of 2-(2′-(*tert*-butyl­thio)­phen­yl)pyridine, see: Clavier *et al.* (2003 ▶). For related structures, see: Shibue *et al.* (2008 ▶); Sau *et al.* (2010 ▶).
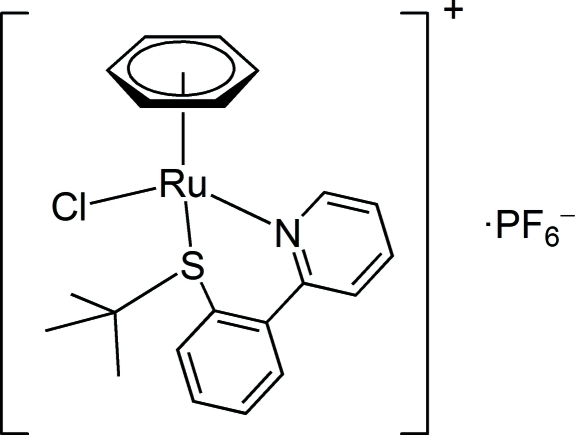


## Experimental

### 

#### Crystal data

[RuCl(C_6_H_6_)(C_15_H_17_NS)]PF_6_
*M* *_r_* = 602.97
Monoclinic, 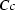

*a* = 16.638 (4) Å
*b* = 10.5589 (19) Å
*c* = 14.327 (3) Å
β = 110.758 (4)°
*V* = 2353.6 (8) Å^3^
*Z* = 4
Mo *K*α radiation
μ = 0.99 mm^−1^
*T* = 193 K
0.24 × 0.17 × 0.09 mm

#### Data collection

Rigaku Mercury diffractometer
Absorption correction: multi-scan (*REQAB*; Jacobson, 1998 ▶) *T*_min_ = 0.725, *T*_max_ = 0.914
11123 measured reflections
4474 independent reflections
4165 reflections with *I* > 2σ(*I*)
*R*_int_ = 0.034

#### Refinement

*R*[*F* ^2^ > 2σ(*F* ^2^)] = 0.037
*wR*(*F* ^2^) = 0.078
*S* = 1.03
4474 reflections
291 parameters
2 restraints
H-atom parameters constrained
Δρ_max_ = 0.52 e Å^−3^
Δρ_min_ = −0.39 e Å^−3^
Absolute structure: Flack (1983 ▶), 1824 Friedel pairs
Flack parameter: 0.03 (3)

### 

Data collection: *CrystalClear* (Rigaku, 1999 ▶); cell refinement: *CrystalClear*; data reduction: *CrystalStructure* (Rigaku/MSC, 2006 ▶); program(s) used to solve structure: *SIR97* (Altomare *et al.*, 1999 ▶); program(s) used to refine structure: *SHELXL97* (Sheldrick, 2008 ▶); molecular graphics: *ORTEP-3 for Windows* (Farrugia, 1997 ▶); software used to prepare material for publication: *CrystalStructure*.

## Supplementary Material

Crystal structure: contains datablocks global, I. DOI: 10.1107/S1600536810049810/fj2364sup1.cif

Structure factors: contains datablocks I. DOI: 10.1107/S1600536810049810/fj2364Isup2.hkl

Additional supplementary materials:  crystallographic information; 3D view; checkCIF report

## Figures and Tables

**Table:** 

Ru1—Cl1	2.3970 (14)
Ru1—S1	2.3671 (10)
Ru1—N1	2.122 (3)
Ru1—C16	2.183 (6)
Ru1—C17	2.187 (6)
Ru1—C18	2.178 (6)
Ru1—C19	2.199 (6)
Ru1—C20	2.172 (7)
Ru1—C21	2.161 (5)

**Table:** 

Cl1—Ru1—S1	93.84 (4)
Cl1—Ru1—N1	86.89 (14)
S1—Ru1—N1	86.76 (9)
Ru1—S1—C11	98.00 (15)
Ru1—S1—C12	123.42 (13)
C11—S1—C12	106.9 (2)
Ru1—N1—C1	116.1 (3)
Ru1—N1—C5	125.4 (3)

**Fig. 1.**
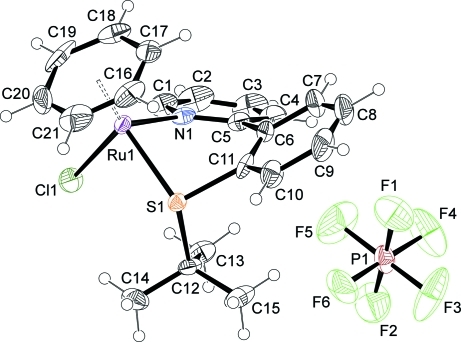
The molecular structure of (I) with numbering scheme. Displacement ellipsoids are drawn at the 40% probability level.

## supplementary crystallographic information

### Comment

Coordination of bidentate ligands bearing thioether sulfur atoms generates chirality at the sulfur center. The chirality of the coordinated S atoms in catalysts would be important in improving enantioselectivities for asymmetric reactions. A variety of chiral *N,S*-bidentate ligands have been developed and used for asymmetric allylic substitution reactions (Mellah *et al.*, 2007). We previously reported the structurally characterized ruthenium(II) arene complex with 4-(2'-pyridyl)dibenzothiophene (PyDBT), [RuCl(PyDBT)(η^6^-C_6_H_6_)]CF_3_SO_3_ (Shibue *et al.*, 2008). In this complex, PyDBT acts as a *N,S*-bidentate ligand to form chiral centers at Ru and S atoms. The pyridine and dibenzothiophene planes in PyDBT are twisted with respect to each other, and the dibenzothiophene moiety is in close proximity to the η^6^-benzene ligand. To clarify the steric interactions around the coordinated S atom, we present here the crystal structure of the title ruthenium(II) arene complex of 2-(2'-(*t*-butylthio)phenyl)pyridine (btppy).

The asymmetric unit of the title compound (I) consists of a [RuCl(btppy)(η^6^-C_6_H_6_)]^+^ cation and a hexafluorophosphate anion (Fig. 1). The Ru center of the complex cation is surrounded by a benzene, a btppy and a chloride ligand to form a three-legged piano-stool structure. The btppy ligand acts as a *N,S*-bidentate ligand to form a six-membered ring. The chelate ring adopts an envelope conformation, which is similar to that in the previously reported ruthenium(II) complex [RuCl(PyDBT)(η^6^-C_6_H_6_)]CF_3_SO_3_, (II) (Shibue *et al.*, 2008). The S—Ru—N bite angle (86.76 (9)°) is larger than that of (II) (79.20 (7)°, 80.00 (7)°, two independent molecules), which is due to the longer C—S bond in the btppy chelate ring (1.820 (5) Å) compared with the PyDBT chelate ring in (II) (C—S = 1.762 (5), 1.760 (4) Å). The Ru—S (2.3671 (10) Å) and Ru—N (2.122 (3) Å) bond lengths in (I) are slightly shorter than those in (II) (Ru—S, 2.3821 (9), 2.3901 (8) Å; Ru—N, 2.161 (3), 2.164 (3) Å), indicating the higher coordinating ability of btppy. The dihedral angle between the pyridine and benzene rings in btppy (39.8 (2)°) is similar to that in PyDBT of (II) (37.7 (2)°).

There are two pair of racemic diastereomers with (*S*_Ru_, *S*_S_) and (*R*_Ru_, *R*_S_) configurations in the unit cell. The *t*-butyl group on the coordinated S atom is placed far from the η^6^-benzene ligand. An average Ru—C distance of 2.180 (6) Å in (I) is comparable to that in (II) (2.192 (4), 2.180 (4) Å). The Cl—Ru—S angle (93.84 (4)°) in (I) is larger than that in (II) (82.84 (3)°, 84.87 (3)°), and no significant interaction was observed between the *t*-butyl group and the chloro ligand. Molecular modeling analysis suggests that the other diastereomers with configurations of (*S*_Ru_, *R*_S_) and (*R*_Ru_, *S*_S_) cause a severe repulsive interaction between the *t*-butyl group and the benzene ligand. The ^1^H NMR spectrum of (I) in CDCl_3_ revealed the presence of a single diastereomer. This suggests that the (*S*_Ru_, *S*_S_) and (*R*_Ru_, *R*_S_) isomers observed in the crystal are retained in solution. Similar stereoselectivity was recently reported for the iridium(III) complex [(η^5^-C_5_Me_5_)Ir(η^2^-ppy-S-*p*-tol)(H_2_O)](OTf)_2_ (Sau *et al.*, 2010), in which the structure of the chelate ring is analogous to that for btppy in (I).

### Experimental

The btppy ligand was prepared according to a literature procedure (Clavier *et al.*, 2003). For the synthesis of the title compound (I), [RuCl_2_(C_6_H_6_)]_2_ (51 mg, 0.10 mmol) was added to a deoxygenated solution of btppy (50 mg, 0.21 mmol) in methanol (10 ml). The reaction mixture was refluxed for 6 h under an argon atmosphere. After cooling to room temperature, KPF_6_ (60 mg, 0.33 mmol) was added. The resulting orange solution was concentrated under reduced pressure. The residue was dissolved in chloroform (10 ml), and the insoluble white solid was removed by filtration. The filtrate was concentrated under reduced pressure, and the resulting brown residue was recrystallized by vapor diffusion of diethyl ether into a dichloromethane solution to afford yellow crystals (19 mg, 15%). Anal. Calcd for C_21_H_23_ClF_6_NPRuS: C, 41.83; H, 3.84; N, 2.32%. Found: C, 41.78; H, 3.82; N, 2.35%. ^1^H NMR (300 MHz, CDCl_3_): δ 1.00 (s, 9H, *t*-Bu), 5.62 (s, 6H, C_6_*H*_6_), 7.51 (ddd, *J* = 7.5, 6.0, 1.4 Hz, 1H), 7.62 (td, *J* = 7.6, 1.2 Hz, 1H), 7.77 (td, *J* = 7.7, 1.2 Hz, 1H), 7.82 (dd, *J* = 7.9, 1.0 Hz, 1H), 7.84 (dd, *J* = 7.6, 1.2 Hz, 1H), 8.02 (m, 2H), 9.39 (dd, *J* = 5.9, 1.2 Hz, 1H).

### Refinement

All non-hydrogen atoms were refined anisotropically. Hydrogen atoms were located on calculated positions with C—H(aromatic) = 0.95 Å and C—H(methyl) = 0.98 Å, and were refined using a riding model with *U*_iso_(H) = 1.2*U*_eq_(C).

### Crystal data

**Table:**

[RuCl(C_6_H_6_)(C_15_H_17_NS)]PF_6_	*F*(000) = 1208.00
*M**_r_* = 602.97	*D*_x_ = 1.702 Mg m^−^^3^
Monoclinic, *C**c*	Mo *K*α radiation, λ = 0.71070 Å
Hall symbol: C -2yc	Cell parameters from 5146 reflections
*a* = 16.638 (4) Å	θ = 4.2–27.5°
*b* = 10.5589 (19) Å	µ = 0.99 mm^−^^1^
*c* = 14.327 (3) Å	*T* = 193 K
β = 110.758 (4)°	Prism, yellow
*V* = 2353.6 (8) Å^3^	0.24 × 0.17 × 0.09 mm
*Z* = 4	

### Data collection

**Table:**

Rigaku Mercury diffractometer	4474 independent reflections
Radiation source: rotating anode X-ray tube	4165 reflections with *F*^2^ > 2σ(*F*^2^)
graphite	*R*_int_ = 0.034
Detector resolution: 7.31 pixels mm^-1^	θ_max_ = 27.5°, θ_min_ = 4.2°
ω scans	*h* = −21→21
Absorption correction: multi-scan (*REQAB*; Jacobson, 1998)	*k* = −13→12
*T*_min_ = 0.725, *T*_max_ = 0.914	*l* = −18→18
11123 measured reflections	

### Refinement

**Table:**

Refinement on *F*^2^	H-atom parameters constrained
Least-squares matrix: full	*w* = 1/[σ^2^(*F*_o_^2^) + (0.0317*P*)^2^ + 6.2745*P*] where *P* = (*F*_o_^2^ + 2*F*_c_^2^)/3
*R*[*F*^2^ > 2σ(*F*^2^)] = 0.037	(Δ/σ)_max_ < 0.001
*wR*(*F*^2^) = 0.078	Δρ_max_ = 0.52 e Å^−^^3^
*S* = 1.03	Δρ_min_ = −0.39 e Å^−^^3^
4474 reflections	Absolute structure: Flack (1983), 1824 Friedel pairs
291 parameters	Flack parameter: 0.03 (3)
2 restraints	

### Special details

**Table:**

Refinement. Refinement was performed using all reflections. The weighted *R*-factor (*wR*) and goodness of fit (*S*) are based on *F*^2^. *R*-factor (gt) are based on *F*. The threshold expression of *F*^2^ > 2.0 σ(*F*^2^) is used only for calculating *R*-factor (gt).

### Fractional atomic coordinates and isotropic or equivalent isotropic displacement parameters (Å^2^)

**Table:**

	*x*	*y*	*z*	*U*_iso_*/*U*_eq_	
Ru1	0.440889 (18)	0.11432 (3)	0.376620 (19)	0.02598 (8)	
Cl1	0.45796 (9)	0.16185 (16)	0.22110 (9)	0.0513 (3)	
S1	0.58842 (7)	0.14331 (10)	0.47160 (8)	0.0254 (2)	
P1	0.69584 (11)	0.73747 (15)	0.57967 (11)	0.0475 (3)	
F1	0.6579 (3)	0.6607 (5)	0.6493 (3)	0.0999 (15)	
F2	0.7309 (4)	0.8098 (5)	0.5069 (4)	0.1156 (18)	
F3	0.7809 (3)	0.7558 (4)	0.6683 (4)	0.131 (2)	
F4	0.6641 (4)	0.8656 (4)	0.6080 (4)	0.120 (2)	
F5	0.6079 (3)	0.7164 (7)	0.4899 (4)	0.138 (2)	
F6	0.7296 (3)	0.6079 (4)	0.5553 (4)	0.1003 (16)	
N1	0.4267 (2)	0.3121 (3)	0.3922 (3)	0.0329 (9)	
C1	0.3753 (3)	0.3726 (5)	0.3113 (4)	0.0462 (12)	
C2	0.3601 (4)	0.5007 (6)	0.3073 (6)	0.0675 (19)	
C3	0.4025 (4)	0.5699 (5)	0.3912 (6)	0.066 (2)	
C4	0.4540 (3)	0.5107 (4)	0.4763 (5)	0.0516 (14)	
C5	0.4655 (3)	0.3802 (4)	0.4765 (3)	0.0336 (10)	
C6	0.5200 (3)	0.3164 (4)	0.5718 (3)	0.0369 (11)	
C7	0.5125 (4)	0.3609 (5)	0.6596 (4)	0.0549 (15)	
C8	0.5601 (5)	0.3089 (7)	0.7504 (4)	0.0643 (18)	
C9	0.6163 (4)	0.2118 (6)	0.7564 (4)	0.0589 (18)	
C10	0.6249 (3)	0.1659 (5)	0.6696 (3)	0.0452 (12)	
C11	0.5760 (3)	0.2207 (4)	0.5792 (3)	0.0306 (10)	
C12	0.6615 (2)	0.2452 (4)	0.4304 (3)	0.0346 (10)	
C13	0.6198 (3)	0.3698 (4)	0.3827 (4)	0.0450 (13)	
C14	0.6825 (3)	0.1590 (6)	0.3568 (4)	0.0515 (14)	
C15	0.7403 (3)	0.2697 (7)	0.5230 (4)	0.0566 (17)	
C16	0.4470 (3)	−0.0321 (5)	0.4865 (4)	0.0507 (15)	
C17	0.3813 (3)	0.0508 (5)	0.4822 (4)	0.0457 (13)	
C18	0.3169 (3)	0.0775 (5)	0.3907 (5)	0.0519 (15)	
C19	0.3172 (4)	0.0179 (7)	0.3037 (4)	0.069 (2)	
C20	0.3868 (5)	−0.0664 (7)	0.3120 (6)	0.076 (2)	
C21	0.4492 (6)	−0.0882 (5)	0.4002 (7)	0.068 (2)	
H1	0.3474	0.3241	0.2528	0.055*	
H2	0.3218	0.5398	0.2487	0.081*	
H3	0.3961	0.6593	0.3903	0.079*	
H4	0.4818	0.5585	0.5351	0.062*	
H7	0.4739	0.4283	0.6569	0.066*	
H8	0.5538	0.3406	0.8095	0.077*	
H9	0.6491	0.1763	0.8193	0.071*	
H10	0.6634	0.0984	0.6720	0.054*	
H13A	0.5695	0.3517	0.3231	0.054*	
H13B	0.6614	0.4197	0.3638	0.054*	
H13C	0.6020	0.4178	0.4306	0.054*	
H14A	0.6306	0.1453	0.2980	0.062*	
H14B	0.7037	0.0775	0.3887	0.062*	
H14C	0.7268	0.1989	0.3362	0.062*	
H15A	0.7247	0.3251	0.5687	0.068*	
H15B	0.7849	0.3106	0.5036	0.068*	
H15C	0.7622	0.1891	0.5563	0.068*	
H16	0.4907	−0.0505	0.5488	0.061*	
H17	0.3800	0.0896	0.5415	0.055*	
H18	0.2728	0.1363	0.3876	0.062*	
H19	0.2728	0.0329	0.2413	0.083*	
H20	0.3888	−0.1077	0.2539	0.091*	
H21	0.4955	−0.1428	0.4034	0.082*	

### Atomic displacement parameters (Å^2^)

**Table:**

	*U*^11^	*U*^22^	*U*^33^	*U*^12^	*U*^13^	*U*^23^
Ru1	0.02434 (16)	0.02938 (15)	0.02221 (15)	−0.00544 (19)	0.00577 (12)	−0.00200 (19)
Cl1	0.0469 (7)	0.0825 (10)	0.0235 (5)	−0.0105 (7)	0.0113 (5)	−0.0022 (6)
S1	0.0239 (5)	0.0271 (5)	0.0237 (5)	−0.0005 (4)	0.0065 (4)	0.0003 (4)
P1	0.0524 (9)	0.0479 (8)	0.0337 (7)	0.0149 (7)	0.0047 (6)	−0.0053 (6)
F1	0.124 (4)	0.100 (3)	0.094 (3)	0.014 (3)	0.062 (3)	0.023 (2)
F2	0.148 (5)	0.094 (3)	0.114 (4)	−0.026 (3)	0.060 (3)	−0.005 (3)
F3	0.113 (4)	0.062 (2)	0.125 (4)	0.001 (2)	−0.071 (3)	0.003 (2)
F4	0.196 (6)	0.076 (3)	0.098 (3)	0.084 (3)	0.064 (4)	0.020 (2)
F5	0.093 (3)	0.205 (6)	0.073 (3)	−0.024 (4)	−0.025 (3)	0.027 (3)
F6	0.120 (3)	0.070 (2)	0.139 (4)	0.001 (2)	0.079 (3)	−0.043 (2)
N1	0.020 (2)	0.036 (2)	0.041 (2)	0.0042 (16)	0.0090 (17)	0.0089 (19)
C1	0.033 (2)	0.043 (3)	0.056 (3)	0.006 (2)	0.009 (2)	0.015 (2)
C2	0.046 (3)	0.058 (4)	0.090 (5)	0.013 (3)	0.014 (3)	0.037 (3)
C3	0.056 (4)	0.027 (2)	0.116 (6)	0.012 (2)	0.031 (4)	0.022 (3)
C4	0.046 (3)	0.028 (2)	0.082 (4)	0.003 (2)	0.025 (3)	−0.002 (2)
C5	0.026 (2)	0.032 (2)	0.042 (2)	−0.0003 (19)	0.012 (2)	0.002 (2)
C6	0.037 (2)	0.038 (2)	0.038 (2)	−0.013 (2)	0.016 (2)	−0.016 (2)
C7	0.066 (4)	0.053 (3)	0.051 (3)	−0.008 (2)	0.027 (3)	−0.025 (2)
C8	0.081 (4)	0.080 (4)	0.036 (3)	−0.025 (3)	0.026 (3)	−0.025 (3)
C9	0.074 (4)	0.074 (4)	0.020 (2)	−0.021 (3)	0.004 (3)	0.000 (2)
C10	0.050 (3)	0.050 (3)	0.029 (2)	−0.003 (2)	0.006 (2)	0.002 (2)
C11	0.035 (2)	0.025 (2)	0.021 (2)	−0.0133 (18)	−0.004 (2)	0.0025 (17)
C12	0.022 (2)	0.048 (2)	0.033 (2)	−0.006 (2)	0.010 (2)	0.005 (2)
C13	0.042 (3)	0.042 (2)	0.051 (3)	−0.008 (2)	0.016 (2)	0.015 (2)
C14	0.046 (3)	0.071 (4)	0.047 (3)	0.001 (2)	0.028 (2)	−0.001 (2)
C15	0.033 (3)	0.079 (4)	0.053 (4)	−0.017 (2)	0.008 (2)	0.008 (3)
C16	0.047 (3)	0.043 (3)	0.055 (3)	−0.017 (2)	0.008 (2)	0.022 (2)
C17	0.052 (3)	0.051 (3)	0.043 (3)	−0.020 (2)	0.027 (2)	0.001 (2)
C18	0.030 (2)	0.047 (3)	0.078 (4)	−0.005 (2)	0.019 (3)	0.018 (3)
C19	0.052 (4)	0.085 (5)	0.044 (3)	−0.049 (3)	−0.014 (3)	0.015 (3)
C20	0.096 (6)	0.066 (4)	0.088 (5)	−0.050 (4)	0.062 (5)	−0.052 (4)
C21	0.062 (5)	0.027 (2)	0.123 (9)	−0.009 (3)	0.042 (6)	0.000 (3)

### Geometric parameters (Å, °)

**Table:**

Ru1—Cl1	2.3970 (14)	C12—C14	1.524 (8)
Ru1—S1	2.3671 (10)	C12—C15	1.521 (6)
Ru1—N1	2.122 (3)	C16—C17	1.386 (8)
Ru1—C16	2.183 (6)	C16—C21	1.383 (12)
Ru1—C17	2.187 (6)	C17—C18	1.396 (7)
Ru1—C18	2.178 (6)	C18—C19	1.398 (10)
Ru1—C19	2.199 (6)	C19—C20	1.432 (12)
Ru1—C20	2.172 (7)	C20—C21	1.340 (11)
Ru1—C21	2.161 (5)	C1—H1	0.950
S1—C11	1.820 (5)	C2—H2	0.950
S1—C12	1.869 (5)	C3—H3	0.950
P1—F1	1.580 (6)	C4—H4	0.950
P1—F2	1.563 (7)	C7—H7	0.950
P1—F3	1.542 (5)	C8—H8	0.950
P1—F4	1.558 (6)	C9—H9	0.950
P1—F5	1.585 (5)	C10—H10	0.950
P1—F6	1.565 (5)	C13—H13A	0.980
N1—C1	1.335 (6)	C13—H13B	0.980
N1—C5	1.357 (6)	C13—H13C	0.980
C1—C2	1.374 (8)	C14—H14A	0.980
C2—C3	1.369 (10)	C14—H14B	0.980
C3—C4	1.368 (9)	C14—H14C	0.980
C4—C5	1.391 (7)	C15—H15A	0.980
C5—C6	1.505 (6)	C15—H15B	0.980
C6—C7	1.389 (9)	C15—H15C	0.980
C6—C11	1.353 (7)	C16—H16	0.950
C7—C8	1.374 (8)	C17—H17	0.950
C8—C9	1.370 (11)	C18—H18	0.950
C9—C10	1.389 (9)	C19—H19	0.950
C10—C11	1.388 (6)	C20—H20	0.950
C12—C13	1.531 (7)	C21—H21	0.950
			
Cl1···C16^i^	3.574 (6)	H2···H14B^xiii^	3.292
S1···F4^ii^	3.502 (5)	H2···H14C^xiii^	2.880
F1···C14^iii^	3.430 (8)	H2···H15C^xii^	3.534
F1···C19^iv^	3.365 (8)	H3···F3^ix^	3.206
F2···C9^v^	3.423 (7)	H3···F5	3.354
F2···C18^vi^	3.538 (9)	H3···C14^xiii^	3.414
F3···C2^vii^	3.233 (8)	H3···C16^viii^	3.522
F3···C19^iv^	3.414 (8)	H3···C20^viii^	3.090
F3···C20^iv^	2.962 (9)	H3···C21^viii^	2.797
F4···S1^viii^	3.502 (5)	H3···H8^v^	3.222
F4···C10^viii^	3.415 (8)	H3···H20^viii^	3.117
F4···C14^iii^	3.476 (9)	H3···H21^viii^	2.630
F4···C16^viii^	3.575 (8)	H3···H14B^xiii^	3.308
F5···C3	3.556 (9)	H3···H14C^xiii^	2.677
F5···C4	3.310 (9)	H3···H15B^xiii^	3.277
F5···C8^v^	3.247 (8)	H4···F1	3.005
F5···C9^v^	3.481 (9)	H4···F5	2.926
F5···C21^viii^	3.236 (10)	H7···C2^iii^	3.418
F6···C13	3.545 (6)	H7···H2^iii^	3.258
F6···C17^vi^	3.116 (9)	H7···H13A^iii^	3.303
F6···C18^vi^	3.194 (10)	H8···F5^iii^	2.490
F6···C19^iv^	3.589 (8)	H8···C3^iii^	3.270
C2···F3^ix^	3.233 (8)	H8···C13^iii^	3.292
C3···F5	3.556 (9)	H8···H3^iii^	3.222
C4···F5	3.310 (9)	H8···H18^iv^	3.419
C8···F5^iii^	3.247 (8)	H8···H20^x^	3.560
C9···F2^iii^	3.423 (7)	H8···H21^x^	2.836
C9···F5^iii^	3.481 (9)	H8···H13A^iii^	3.259
C10···F4^ii^	3.415 (8)	H8···H13B^iii^	3.037
C13···F6	3.545 (6)	H8···H13C^iii^	3.028
C14···F1^v^	3.430 (8)	H9···F2^iii^	2.550
C14···F4^v^	3.476 (9)	H9···F5^iii^	2.986
C16···Cl1^x^	3.574 (6)	H9···C14^x^	3.595
C16···F4^ii^	3.575 (8)	H9···H18^iv^	2.772
C17···F6^xi^	3.116 (9)	H9···H21^x^	3.207
C18···F2^xi^	3.538 (9)	H9···H14A^x^	3.413
C18···F6^xi^	3.194 (10)	H9···H14B^x^	2.891
C19···F1^xii^	3.365 (8)	H10···F4^ii^	2.626
C19···F3^xii^	3.414 (8)	H10···C1^iv^	3.394
C19···F6^xii^	3.589 (8)	H10···C2^iv^	3.328
C20···F3^xii^	2.962 (9)	H10···H1^iv^	2.979
C21···F5^ii^	3.236 (10)	H10···H2^iv^	2.869
Cl1···H16^i^	2.954	H10···H14A^x^	3.299
Cl1···H15B^xii^	3.425	H10···H14B^x^	3.474
P1···H2^vii^	3.491	H16···Cl1^x^	2.954
P1···H18^vi^	3.584	H16···F4^ii^	2.845
P1···H20^iv^	3.569	H16···F5^ii^	3.425
P1···H14C^iii^	3.589	H16···H15B^xi^	3.567
F1···H4	3.005	H17···F2^xi^	3.307
F1···H19^iv^	2.790	H17···F3^xi^	3.351
F1···H13A^iii^	3.313	H17···F6^xi^	2.588
F1···H13B^iii^	3.170	H17···H20^x^	3.002
F1···H14A^iii^	3.103	H17···H15B^xi^	3.296
F1···H14C^iii^	2.916	H18···P1^xi^	3.584
F2···H9^v^	2.550	H18···F2^xi^	2.758
F2···H17^vi^	3.307	H18···F6^xi^	2.757
F2···H18^vi^	2.758	H18···C8^xii^	3.434
F2···H14B^viii^	3.243	H18···C9^xii^	3.068
F3···H2^vii^	2.428	H18···H8^xii^	3.419
F3···H3^vii^	3.206	H18···H9^xii^	2.772
F3···H17^vi^	3.351	H18···H13B^xi^	2.887
F3···H19^iv^	3.241	H19···F1^xii^	2.790
F3···H20^iv^	2.369	H19···F3^xii^	3.241
F3···H14C^iii^	2.892	H19···F6^xii^	2.910
F4···H2^vii^	2.863	H19···H13B^xi^	3.202
F4···H10^viii^	2.626	H19···H15A^xii^	2.757
F4···H16^viii^	2.845	H20···P1^xii^	3.569
F4···H21^viii^	3.262	H20···F3^xii^	2.369
F4···H14A^iii^	2.968	H20···F6^xii^	3.127
F4···H14C^iii^	3.133	H20···C8^i^	3.569
F5···H3	3.354	H20···H3^ii^	3.117
F5···H4	2.926	H20···H8^i^	3.560
F5···H8^v^	2.490	H20···H17^i^	3.002
F5···H9^v^	2.986	H21···F4^ii^	3.262
F5···H16^viii^	3.425	H21···F5^ii^	2.366
F5···H21^viii^	2.366	H21···C3^ii^	3.382
F5···H13C	3.258	H21···C8^i^	3.272
F6···H17^vi^	2.588	H21···C9^i^	3.465
F6···H18^vi^	2.757	H21···H3^ii^	2.630
F6···H19^iv^	2.910	H21···H8^i^	2.836
F6···H20^iv^	3.127	H21···H9^i^	3.207
F6···H13B	3.249	H13A···F1^v^	3.313
F6···H13C	3.006	H13A···H7^v^	3.303
F6···H15A	2.996	H13A···H8^v^	3.259
F6···H15B	3.425	H13B···F1^v^	3.170
C1···H10^xii^	3.394	H13B···F6	3.249
C1···H18	3.414	H13B···C8^v^	3.428
C1···H13A	3.183	H13B···C18^vi^	2.986
C1···H13C	3.579	H13B···C19^vi^	3.181
C1···H15C^xii^	3.527	H13B···H8^v^	3.037
C2···H7^v^	3.418	H13B···H18^vi^	2.887
C2···H10^xii^	3.328	H13B···H19^vi^	3.202
C2···H14B^xiii^	3.308	H13C···F5	3.258
C2···H14C^xiii^	3.182	H13C···F6	3.006
C3···H8^v^	3.270	H13C···H8^v^	3.028
C3···H21^viii^	3.382	H14A···F1^v^	3.103
C3···H14B^xiii^	3.296	H14A···F4^v^	2.968
C3···H14C^xiii^	3.065	H14A···H9^i^	3.413
C8···H18^iv^	3.434	H14A···H10^i^	3.299
C8···H20^x^	3.569	H14B···F2^ii^	3.243
C8···H21^x^	3.272	H14B···C2^xiv^	3.308
C8···H13B^iii^	3.428	H14B···C3^xiv^	3.296
C9···H18^iv^	3.068	H14B···H2^xiv^	3.292
C9···H21^x^	3.465	H14B···H3^xiv^	3.308
C10···H1^iv^	3.466	H14B···H9^i^	2.891
C13···H8^v^	3.292	H14B···H10^i^	3.474
C14···H2^xiv^	3.449	H14C···P1^v^	3.589
C14···H3^xiv^	3.414	H14C···F1^v^	2.916
C14···H9^i^	3.595	H14C···F3^v^	2.892
C15···H1^iv^	3.293	H14C···F4^v^	3.133
C16···H3^ii^	3.522	H14C···C2^xiv^	3.182
C16···H15B^xi^	3.250	H14C···C3^xiv^	3.065
C17···H15B^xi^	3.074	H14C···H2^xiv^	2.880
C18···H13B^xi^	2.986	H14C···H3^xiv^	2.677
C18···H15B^xi^	3.384	H15A···F6	2.996
C19···H13B^xi^	3.181	H15A···C19^iv^	3.575
C19···H15A^xii^	3.575	H15A···H1^iv^	3.129
C20···H3^ii^	3.090	H15A···H19^iv^	2.757
C21···H3^ii^	2.797	H15B···Cl1^iv^	3.425
H1···C10^xii^	3.466	H15B···F6	3.425
H1···C15^xii^	3.293	H15B···C16^vi^	3.250
H1···H10^xii^	2.979	H15B···C17^vi^	3.074
H1···H15A^xii^	3.129	H15B···C18^vi^	3.384
H1···H15C^xii^	2.671	H15B···H3^xiv^	3.277
H2···P1^ix^	3.491	H15B···H16^vi^	3.567
H2···F3^ix^	2.428	H15B···H17^vi^	3.296
H2···F4^ix^	2.863	H15C···C1^iv^	3.527
H2···C14^xiii^	3.449	H15C···H1^iv^	2.671
H2···H7^v^	3.258	H15C···H2^iv^	3.534
H2···H10^xii^	2.869		
			
Cl1—Ru1—S1	93.84 (4)	S1—C12—C14	102.1 (3)
Cl1—Ru1—N1	86.89 (14)	S1—C12—C15	106.2 (4)
Cl1—Ru1—C16	145.87 (18)	C13—C12—C14	112.5 (4)
Cl1—Ru1—C17	159.90 (13)	C13—C12—C15	110.9 (4)
Cl1—Ru1—C18	123.49 (17)	C14—C12—C15	111.5 (4)
Cl1—Ru1—C19	93.14 (19)	Ru1—C16—C17	71.7 (3)
Cl1—Ru1—C20	87.4 (2)	Ru1—C16—C21	70.6 (4)
Cl1—Ru1—C21	109.3 (3)	C17—C16—C21	120.1 (5)
S1—Ru1—N1	86.76 (9)	Ru1—C17—C16	71.4 (4)
S1—Ru1—C16	84.44 (15)	Ru1—C17—C18	71.0 (4)
S1—Ru1—C17	105.91 (13)	C16—C17—C18	120.0 (5)
S1—Ru1—C18	142.48 (17)	Ru1—C18—C17	71.7 (3)
S1—Ru1—C19	159.70 (19)	Ru1—C18—C19	72.2 (4)
S1—Ru1—C20	123.2 (2)	C17—C18—C19	120.1 (5)
S1—Ru1—C21	92.4 (2)	Ru1—C19—C18	70.5 (3)
N1—Ru1—C16	126.9 (2)	Ru1—C19—C20	69.8 (4)
N1—Ru1—C17	97.7 (2)	C18—C19—C20	117.7 (5)
N1—Ru1—C18	91.25 (19)	Ru1—C20—C19	71.9 (4)
N1—Ru1—C19	112.7 (2)	Ru1—C20—C21	71.5 (4)
N1—Ru1—C20	149.9 (2)	C19—C20—C21	121.2 (8)
N1—Ru1—C21	163.8 (3)	Ru1—C21—C16	72.3 (3)
C16—Ru1—C17	37.0 (2)	Ru1—C21—C20	72.4 (4)
C16—Ru1—C18	67.1 (2)	C16—C21—C20	120.8 (8)
C16—Ru1—C19	79.1 (2)	N1—C1—H1	118.0
C16—Ru1—C20	65.9 (2)	C2—C1—H1	118.0
C16—Ru1—C21	37.1 (3)	C1—C2—H2	121.3
C17—Ru1—C18	37.3 (2)	C3—C2—H2	121.3
C17—Ru1—C19	67.0 (2)	C2—C3—H3	119.9
C17—Ru1—C20	78.7 (3)	C4—C3—H3	119.9
C17—Ru1—C21	67.0 (3)	C3—C4—H4	120.1
C18—Ru1—C19	37.2 (2)	C5—C4—H4	120.1
C18—Ru1—C20	67.7 (3)	C6—C7—H7	119.5
C18—Ru1—C21	79.6 (3)	C8—C7—H7	119.5
C19—Ru1—C20	38.2 (3)	C7—C8—H8	119.7
C19—Ru1—C21	67.3 (3)	C9—C8—H8	119.7
C20—Ru1—C21	36.0 (3)	C8—C9—H9	120.3
Ru1—S1—C11	98.00 (15)	C10—C9—H9	120.3
Ru1—S1—C12	123.42 (13)	C9—C10—H10	120.7
C11—S1—C12	106.9 (2)	C11—C10—H10	120.7
F1—P1—F2	177.5 (2)	C12—C13—H13A	109.5
F1—P1—F3	90.0 (3)	C12—C13—H13B	109.5
F1—P1—F4	91.8 (3)	C12—C13—H13C	109.5
F1—P1—F5	88.8 (3)	H13A—C13—H13B	109.5
F1—P1—F6	87.0 (3)	H13A—C13—H13C	109.5
F2—P1—F3	92.1 (3)	H13B—C13—H13C	109.5
F2—P1—F4	89.5 (3)	C12—C14—H14A	109.5
F2—P1—F5	89.0 (3)	C12—C14—H14B	109.5
F2—P1—F6	91.7 (3)	C12—C14—H14C	109.5
F3—P1—F4	88.5 (2)	H14A—C14—H14B	109.5
F3—P1—F5	178.8 (3)	H14A—C14—H14C	109.5
F3—P1—F6	89.8 (2)	H14B—C14—H14C	109.5
F4—P1—F5	91.8 (3)	C12—C15—H15A	109.5
F4—P1—F6	177.9 (2)	C12—C15—H15B	109.5
F5—P1—F6	89.8 (3)	C12—C15—H15C	109.5
Ru1—N1—C1	116.1 (3)	H15A—C15—H15B	109.5
Ru1—N1—C5	125.4 (3)	H15A—C15—H15C	109.5
C1—N1—C5	118.4 (4)	H15B—C15—H15C	109.5
N1—C1—C2	124.0 (5)	Ru1—C16—H16	130.3
C1—C2—C3	117.3 (6)	C17—C16—H16	120.0
C2—C3—C4	120.3 (5)	C21—C16—H16	120.0
C3—C4—C5	119.8 (5)	Ru1—C17—H17	130.2
N1—C5—C4	120.1 (4)	C16—C17—H17	120.0
N1—C5—C6	121.0 (4)	C18—C17—H17	120.0
C4—C5—C6	118.9 (4)	Ru1—C18—H18	128.4
C5—C6—C7	116.9 (4)	C17—C18—H18	119.9
C5—C6—C11	125.6 (5)	C19—C18—H18	119.9
C7—C6—C11	117.5 (4)	Ru1—C19—H19	130.9
C6—C7—C8	121.1 (6)	C18—C19—H19	121.2
C7—C8—C9	120.6 (6)	C20—C19—H19	121.2
C8—C9—C10	119.3 (5)	Ru1—C20—H20	129.7
C9—C10—C11	118.5 (5)	C19—C20—H20	119.4
S1—C11—C6	123.5 (3)	C21—C20—H20	119.4
S1—C11—C10	113.5 (3)	Ru1—C21—H21	127.9
C6—C11—C10	122.9 (5)	C16—C21—H21	119.6
S1—C12—C13	113.2 (3)	C20—C21—H21	119.6
			
Cl1—Ru1—S1—C11	141.23 (14)	C18—Ru1—C17—C16	132.5 (5)
Cl1—Ru1—S1—C12	24.8 (2)	C17—Ru1—C19—C18	29.6 (3)
Cl1—Ru1—N1—C1	45.1 (4)	C17—Ru1—C19—C20	−101.3 (5)
Cl1—Ru1—N1—C5	−134.7 (4)	C19—Ru1—C17—C16	102.9 (3)
Cl1—Ru1—C16—C17	−145.4 (2)	C19—Ru1—C17—C18	−29.6 (3)
Cl1—Ru1—C16—C21	−12.8 (6)	C17—Ru1—C20—C19	67.0 (4)
Cl1—Ru1—C17—C16	112.0 (4)	C17—Ru1—C20—C21	−66.1 (6)
Cl1—Ru1—C17—C18	−20.4 (6)	C20—Ru1—C17—C16	64.7 (3)
Cl1—Ru1—C18—C17	171.7 (2)	C20—Ru1—C17—C18	−67.8 (3)
Cl1—Ru1—C18—C19	40.4 (4)	C17—Ru1—C21—C16	−28.8 (4)
Cl1—Ru1—C19—C18	−147.3 (3)	C17—Ru1—C21—C20	102.9 (6)
Cl1—Ru1—C19—C20	81.9 (4)	C21—Ru1—C17—C16	28.9 (3)
Cl1—Ru1—C20—C19	−98.3 (4)	C21—Ru1—C17—C18	−103.6 (4)
Cl1—Ru1—C20—C21	128.5 (6)	C18—Ru1—C19—C20	−130.9 (6)
Cl1—Ru1—C21—C16	172.4 (3)	C19—Ru1—C18—C17	131.4 (5)
Cl1—Ru1—C21—C20	−55.9 (7)	C18—Ru1—C20—C19	29.6 (4)
S1—Ru1—N1—C1	139.1 (4)	C18—Ru1—C20—C21	−103.5 (7)
S1—Ru1—N1—C5	−40.7 (4)	C20—Ru1—C18—C17	101.0 (4)
N1—Ru1—S1—C11	54.57 (19)	C20—Ru1—C18—C19	−30.4 (4)
N1—Ru1—S1—C12	−61.8 (2)	C18—Ru1—C21—C16	−65.6 (4)
S1—Ru1—C16—C17	126.0 (3)	C18—Ru1—C21—C20	66.1 (6)
S1—Ru1—C16—C21	−101.5 (4)	C21—Ru1—C18—C17	65.4 (4)
C16—Ru1—S1—C11	−73.0 (2)	C21—Ru1—C18—C19	−66.0 (4)
C16—Ru1—S1—C12	170.6 (2)	C19—Ru1—C20—C21	−133.1 (9)
S1—Ru1—C17—C16	−56.9 (3)	C20—Ru1—C19—C18	130.9 (6)
S1—Ru1—C17—C18	170.7 (3)	C19—Ru1—C21—C16	−102.4 (5)
C17—Ru1—S1—C11	−42.6 (2)	C19—Ru1—C21—C20	29.3 (6)
C17—Ru1—S1—C12	−159.0 (2)	C21—Ru1—C19—C18	103.2 (5)
S1—Ru1—C18—C17	−14.9 (4)	C21—Ru1—C19—C20	−27.7 (5)
S1—Ru1—C18—C19	−146.2 (3)	C20—Ru1—C21—C16	−131.7 (9)
C18—Ru1—S1—C11	−33.3 (3)	C21—Ru1—C20—C19	133.1 (9)
C18—Ru1—S1—C12	−149.7 (3)	Ru1—S1—C11—C6	−46.4 (4)
S1—Ru1—C19—C18	102.8 (6)	Ru1—S1—C11—C10	129.0 (3)
S1—Ru1—C19—C20	−28.1 (8)	Ru1—S1—C12—C13	41.4 (4)
C19—Ru1—S1—C11	−108.9 (6)	Ru1—S1—C12—C14	−79.8 (3)
C19—Ru1—S1—C12	134.6 (6)	Ru1—S1—C12—C15	163.3 (3)
S1—Ru1—C20—C19	168.7 (3)	C11—S1—C12—C13	−70.6 (3)
S1—Ru1—C20—C21	35.6 (7)	C11—S1—C12—C14	168.2 (2)
C20—Ru1—S1—C11	−129.3 (3)	C11—S1—C12—C15	51.3 (4)
C20—Ru1—S1—C12	114.3 (3)	C12—S1—C11—C6	82.3 (4)
S1—Ru1—C21—C16	77.5 (4)	C12—S1—C11—C10	−102.3 (4)
S1—Ru1—C21—C20	−150.8 (6)	Ru1—N1—C1—C2	−178.6 (6)
C21—Ru1—S1—C11	−109.3 (3)	Ru1—N1—C5—C4	177.3 (4)
C21—Ru1—S1—C12	134.3 (3)	Ru1—N1—C5—C6	−3.9 (7)
N1—Ru1—C16—C17	44.3 (3)	C1—N1—C5—C4	−2.5 (8)
N1—Ru1—C16—C21	176.8 (4)	C1—N1—C5—C6	176.3 (5)
C16—Ru1—N1—C1	−140.3 (4)	C5—N1—C1—C2	1.2 (10)
C16—Ru1—N1—C5	39.9 (5)	N1—C1—C2—C3	1.7 (12)
N1—Ru1—C17—C16	−145.7 (3)	C1—C2—C3—C4	−3.3 (12)
N1—Ru1—C17—C18	81.8 (3)	C2—C3—C4—C5	2.1 (12)
C17—Ru1—N1—C1	−115.3 (4)	C3—C4—C5—N1	0.9 (10)
C17—Ru1—N1—C5	64.9 (4)	C3—C4—C5—C6	−177.9 (6)
N1—Ru1—C18—C17	−101.2 (3)	N1—C5—C6—C7	−140.0 (5)
N1—Ru1—C18—C19	127.5 (3)	N1—C5—C6—C11	40.7 (8)
C18—Ru1—N1—C1	−78.4 (4)	C4—C5—C6—C7	38.8 (8)
C18—Ru1—N1—C5	101.8 (4)	C4—C5—C6—C11	−140.5 (6)
N1—Ru1—C19—C18	−59.3 (4)	C5—C6—C7—C8	−179.6 (6)
N1—Ru1—C19—C20	169.8 (4)	C5—C6—C11—S1	−5.3 (7)
C19—Ru1—N1—C1	−47.0 (5)	C5—C6—C11—C10	179.7 (5)
C19—Ru1—N1—C5	133.2 (4)	C7—C6—C11—S1	175.4 (4)
N1—Ru1—C20—C19	−19.0 (8)	C7—C6—C11—C10	0.5 (8)
N1—Ru1—C20—C21	−152.1 (6)	C11—C6—C7—C8	−0.3 (8)
C20—Ru1—N1—C1	−34.4 (8)	C6—C7—C8—C9	0.1 (8)
C20—Ru1—N1—C5	145.8 (6)	C7—C8—C9—C10	−0.1 (9)
N1—Ru1—C21—C16	−9.2 (12)	C8—C9—C10—C11	0.2 (7)
N1—Ru1—C21—C20	122.5 (8)	C9—C10—C11—S1	−175.8 (5)
C21—Ru1—N1—C1	−133.4 (9)	C9—C10—C11—C6	−0.4 (8)
C21—Ru1—N1—C5	46.8 (11)	Ru1—C16—C17—C18	53.7 (5)
C16—Ru1—C17—C18	−132.5 (5)	Ru1—C16—C21—C20	−56.0 (7)
C17—Ru1—C16—C21	132.6 (5)	C17—C16—C21—Ru1	53.9 (6)
C16—Ru1—C18—C17	28.8 (3)	C17—C16—C21—C20	−2.1 (11)
C16—Ru1—C18—C19	−102.6 (4)	C21—C16—C17—Ru1	−53.4 (6)
C18—Ru1—C16—C17	−29.0 (3)	C21—C16—C17—C18	0.3 (8)
C18—Ru1—C16—C21	103.5 (5)	Ru1—C17—C18—C19	55.7 (6)
C16—Ru1—C19—C18	66.3 (3)	C16—C17—C18—Ru1	−53.9 (5)
C16—Ru1—C19—C20	−64.6 (5)	C16—C17—C18—C19	1.8 (9)
C19—Ru1—C16—C17	−66.0 (3)	Ru1—C18—C19—C20	53.3 (6)
C19—Ru1—C16—C21	66.6 (5)	C17—C18—C19—Ru1	−55.4 (5)
C16—Ru1—C20—C19	103.6 (5)	C17—C18—C19—C20	−2.2 (10)
C16—Ru1—C20—C21	−29.6 (6)	Ru1—C19—C20—C21	54.0 (7)
C20—Ru1—C16—C17	−103.8 (4)	C18—C19—C20—Ru1	−53.6 (6)
C20—Ru1—C16—C21	28.8 (5)	C18—C19—C20—C21	0.4 (11)
C16—Ru1—C21—C20	131.7 (9)	Ru1—C20—C21—C16	55.9 (6)
C21—Ru1—C16—C17	−132.6 (5)	C19—C20—C21—Ru1	−54.2 (7)
C17—Ru1—C18—C19	−131.4 (5)	C19—C20—C21—C16	1.7 (13)

Symmetry codes: (i) *x*, −*y*, *z*−1/2; (ii) *x*, *y*−1, *z*; (iii) *x*, −*y*+1, *z*+1/2; (iv) *x*+1/2, −*y*+1/2, *z*+1/2; (v) *x*, −*y*+1, *z*−1/2; (vi) *x*+1/2, *y*+1/2, *z*; (vii) *x*+1/2, −*y*+3/2, *z*+1/2; (viii) *x*, *y*+1, *z*; (ix) *x*−1/2, −*y*+3/2, *z*−1/2; (x) *x*, −*y*, *z*+1/2; (xi) *x*−1/2, *y*−1/2, *z*; (xii) *x*−1/2, −*y*+1/2, *z*−1/2; (xiii) *x*−1/2, *y*+1/2, *z*; (xiv) *x*+1/2, *y*−1/2, *z*.
